# Longitudinal Effects of Glecaprevir/Pibrentasvir on Liver Function, Fibrosis, and Hepatocellular Carcinoma Risk in Chronic Hepatitis C: A Prospective Multicenter Cohort Study

**DOI:** 10.3390/medicina61091601

**Published:** 2025-09-04

**Authors:** Jung Hee Kim, Jae Hyun Yoon, Sung-Eun Kim, Ji-Won Park, Yewan Park, Gi-Ae Kim, Seong Kyun Na, Young-Sun Lee, Jeong Han Kim

**Affiliations:** 1 Department of Internal Medicine, Hallym University College of Medicine, Dongtan Sacred Heart Hospital, Hwasung 14068, Republic of Korea; mazyyang5@gmail.com; 2 Department of Gastroenterology and Hepatology, Chonnam National University Hospital, College of Medicine, Gwangju 61469, Republic of Korea; zenmake14@gmail.com; 3 Department of Internal Medicine, Hallym University College of Medicine, Anyang 14068, Republic of Korea; sekim@hallym.or.kr (S.-E.K.); miunorijw@hallym.or.kr (J.-W.P.); 4 Department of Internal Medicine, Kyung Hee University School of Medicine, Seoul 02447, Republic of Korea; yewanish@gmail.com (Y.P.); antiankle@hanmail.net (G.-A.K.); 5 Department of Internal Medicine, Inje University Sanggye Paik Hospital, Seoul 01757, Republic of Korea; drcoramdeo@naver.com; 6 Department of Internal Medicine, Korea University Medical Center, Seoul 08308, Republic of Korea; lys810@korea.ac.kr; 7 Department of Internal Medicine, Konkuk University School of Medicine, Seoul 05030, Republic of Korea; 8 Research Institute of Medical Science, Konkuk University School of Medicine, Seoul 05029, Republic of Korea

**Keywords:** hepatitis C, direct-acting antiviral, sustained virologic response, hepatocellular carcinoma, glecaprevir/pibrentasvir

## Abstract

*Background and Aims*: Glecaprevir/pibrentasvir achieves sustained virologic response (SVR) rates above 95% in chronic hepatitis C (CHC). Nevertheless, the residual risk of hepatocellular carcinoma (HCC) after SVR, especially in patients with advanced liver disease, has not been fully defined. We prospectively evaluated longitudinal changes in liver function and fibrosis and sought predictors of post-SVR HCC in a real-world multicenter cohort. *Methods*: A total of 395 CHC patients who attained SVR with glecaprevir/pibrentasvir were followed prospectively. Liver function tests, noninvasive fibrosis indices, and clinical outcomes were recorded at predefined intervals. Cox proportional hazards regression identified factors associated with incident HCC. *Results*: Over a median follow-up of 31.1 months, HCC occurred in 16 patients (4.1%). From univariate analysis, baseline FIB-4 > 3.25, APRI > 1.5, MELD ≥ 10, Child–Pugh score ≥ 6, and clinically significant portal hypertension were associated with HCC. Multivariate analysis retained FIB-4 > 3.25 (*p* = 0.003) and MELD ≥ 10 (*p* = 0.032) as independent predictors. Cumulative incidence rose stepwise with the number of risk factors. *Conclusions*: Despite a virologic cure, patients with advanced fibrosis or impaired liver function remain susceptible to HCC. Risk stratification using FIB-4 and MELD and continued surveillance are therefore warranted.

## 1. Introduction

Chronic hepatitis C virus (HCV) infection affects an estimated 58 million individuals worldwide and remains a major global health burden. Without treatment, progressive hepatic inflammation can lead to cirrhosis and hepatocellular carcinoma (HCC), conditions that drive most liver-related morbidity and mortality [1,2]. The advent of direct-acting antivirals (DAAs) has transformed HCV care, producing SVR rates exceeding 95% and markedly lowering the incidence of hepatic complications [3,4,5]. Among the available DAAs, glecaprevir combined with pibrentasvir offers pan-genotypic coverage and favorable safety, even in patients with compensated cirrhosis or previous treatment failure [6].

Although sustained virologic response (SVR) markedly reduces HCC risk, it does not eliminate it, especially in individuals with advanced fibrosis or cirrhosis [7,8,9]. Consequently, vigilant post-treatment surveillance is mandatory. Several previous studies have documented improved hepatic function and fibrosis regression after direct-acting antivirals (DAAs); however, longitudinal trajectories of these parameters in routine clinical practice are still not fully characterized [10,11]. Defining clinical predictors of post-SVR HCC would allow targeted surveillance and improved long-term outcomes.

We, therefore, aimed to (i) evaluate longitudinal changes in hepatic function and fibrosis indices in patients with chronic HCV infection who received glecaprevir/pibrentasvir and achieved SVR, and (ii) identify factors associated with incident HCC after SVR. The findings will inform risk-stratified surveillance and optimize post-SVR management.

## 2. Materials and Methods

### 2.1. Study Design and Population

This prospective, multicenter observational cohort enrolled patients from six university-affiliated tertiary hospitals in South Korea between July 2018 and December 2024. Eligible participants were adults (≥18 years) with chronic hepatitis C who received glecaprevir/pibrentasvir and achieved SVR, defined as undetectable HCV RNA 12 weeks after treatment completion (Supplementary Figure S1). Exclusion criteria were as follows: (1) inability or refusal to provide written informed consent; (2) patients with a prior diagnosis or imaging evidence of HCC before study enrollment.; (3) co-infection with hepatitis B virus (HBV) or human immunodeficiency virus (HIV); (4) previous liver transplantation; (5) prior antiviral therapy for HCV. The study adhered to the Declaration of Helsinki and was approved by the institutional review boards of all participating centers. All subjects gave written informed consent.

Cirrhosis was diagnosed using a composite assessment that integrated histopathology, imaging features (nodular liver surface, splenomegaly, ascites, or esophagogastric varices), and clinical evidence of hepatic decompensation. The albumin–bilirubin (ALBI) grade, an objective index derived from serum albumin and total bilirubin, was used to quantify hepatic reserve; patients were categorized as grade 1, 2, or 3 using established thresholds [12]. Hepatic fibrosis was assessed noninvasively with the aspartate aminotransferase-to-platelet ratio index (APRI), the fibrosis-4 (FIB-4) index, and liver stiffness measurement (LSM) by transient elastography (FibroScan^®^, Echosens, Paris, France)**.** All laboratory parameters used in the formulas (AST, ALT, albumin, bilirubin, and platelet count) were obtained at the time of chronic hepatitis C diagnosis (baseline), at SVR12, and annually thereafter. Liver stiffness measurements (LSMs) were obtained at SVR12 and annually thereafter. The units of measurement (AST and ALT in IU/L, albumin in g/dL, and total bilirubin in mg/dL) were consistent with those reported in Table 1. Indices were calculated as follows: APRI = [(AST(IU)/upper limit of normal)/platelet count (10^9^/L)] × 100; FIB-4 = [Age (years) × AST (IU/L)]/[platelet count (10^9^/L) × √ALT (IU/L)]; and ALBI = (log_10_ total bilirubin [mg/dL] × 1.494) − (albumin [g/dL] × 0.378). LSM was measured in kilopascals (kPa) using transient elastography (FibroScan^®^), with ≥10 valid measurements and an interquartile range-to-median ratio <0.3 considered reliable.

The composite primary outcome was the first occurrence of HCC or all-cause mortality. Baseline portal hypertension was defined by ascites, esophagogastric varices, or hepatic encephalopathy. Participants underwent semiannual or annual assessments, including laboratory tests (liver biochemistry, serum HCV RNA, and alpha-fetoprotein) and imaging studies (abdominal ultrasound or computed tomography), to track disease progression and identify incident malignancies.

### 2.2. Statistical Analysis

Baseline demographic and clinical variables were summarized descriptively. Continuous data are reported as medians (interquartile range), and categorical data as numbers (percentage). Between-group comparisons employed the Mann–Whitney U test for continuous variables and the Chi-square or Fisher’s exact test for categorical variables, as appropriate. Temporal changes in hepatic biochemical markers and noninvasive fibrosis scores (APRI, FIB-4) were examined with the Wilcoxon signed-rank test. The cumulative incidence of HCC was depicted by Kaplan–Meier curves and compared with the log-rank test. Factors associated with HCC development were investigated using Cox proportional hazards models; variables with *p* < 0.05 in univariate analysis entered the multivariable model through forward stepwise selection. All tests were two-sided, and *p* < 0.05 denoted statistical significance. Analyses were performed with IBM SPSS Statistics, version 27.0 (IBM Corp., Armonk, NY, USA).

Longitudinal trajectories of liver function indices (CTP, MELD, APRI, and FIB-4) were evaluated with linear regression models for repeated measures. Time was treated as categorical (baseline, SVR, and annually to year 5). At each time point, cross-sectional differences between patients with and without HCC were assessed with the Wilcoxon rank-sum test. Adjusted marginal means and 95% confidence intervals were generated with the margins command in Stata/SE version 16, and trajectories were visualized with marginsplot.

## 3. Results

### 3.1. Baseline Characteristics

Of the 395 patients who attained SVR, 16 (4.1%) developed HCC during a median follow-up of 31.1 months. Baseline characteristics stratified by HCC status are summarized in Table 1. Patients who later developed HCC were older than their counterparts without HCC (67.8 ± 9.2 vs. 60.1 ± 12.8 years; *p* = 0.018). Sex distribution, body mass index, HCV genotype, and alcohol intake were comparable between groups.

Compared with non-HCC patients, those who developed HCC had lower platelet counts (115.1 ± 53.9 vs. 187.7 ± 65.0 × 10^9^/L; *p* < 0.001) and higher total bilirubin concentrations (1.02 ± 0.52 vs. 0.74 ± 0.34 mg/dL; *p* = 0.002). Albumin levels did not differ (*p* = 0.964). An ALBI grade ≥ 2 was more common in the HCC cohort (20.0% vs. 13.2%; *p* < 0.001). Markers of advanced liver disease—portal hypertension (18.8% vs. 2.9%; *p* < 0.0001), cirrhosis (87.5% vs. 20.8%; *p* < 0.0001), Child–Pugh class B/C (40.0% vs. 4.3%; *p* < 0.001), and higher MELD scores (8.92 ± 2.66 vs. 7.20 ± 2.54; *p* = 0.017)—were likewise enriched in the HCC group (Supplementary Figure S2).

### 3.2. Longitudinal Changes in Liver Function after Antiviral Treatment

**Figure 1:**
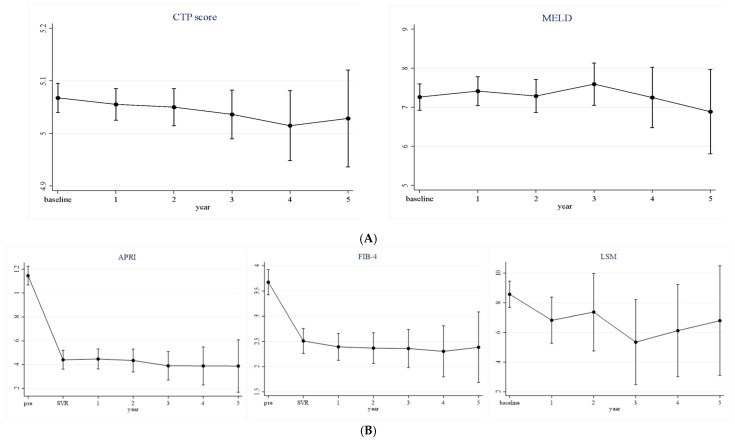
Longitudinal changes in liver function (**A**) and liver fibrosis (**B**) among patients treated with glecaprevir–pibrentasvir therapy (*n* = 395).

To evaluate longitudinal liver function changes, Child–Pugh and MELD scores were calculated at predefined intervals after SVRs (Table 2). Among 395 patients receiving glecaprevir/pibrentasvir, the mean Child–Pugh score at SVR was 5.068 (95% CI, 5.040–5.095) and remained unchanged during the subsequent 5 years. The MELD score at SVR was likewise 7.263 (95% CI, 6.927–7.599) and showed no significant variation at any later visit (Figure 1). Overall, both scores remained stable throughout follow-up.

When stratified by subsequent HCC development, Child–Pugh and MELD scores remained stable within each subgroup over the 5-year period (Supplementary Table S1). Nevertheless, at most assessed time points, both scores were significantly higher in patients who developed HCC than in those who did not (Supplementary Figure S3).

### 3.3. Longitudinal Changes in Liver Fibrosis after Antiviral Treatment

Fibrosis was assessed by APRI, FIB-4, and liver stiffness measurement (LSM) in the same 395 patients (Table 3). APRI and FIB-4 fell markedly from baseline to SVR and continued to decline over the 5-year follow-up, with all post-baseline values remaining statistically lower than baseline (all *p* < 0.001). The mean APRI decreased from 1.146 (95% CI, 1.068–1.224) at baseline to 0.389 (95% CI, 0.168–0.609) at year 5, whereas the FIB-4 index dropped from 3.673 (95% CI, 3.424–3.922) to 2.384 (95% CI, 1.684–3.085). LSM values also declined—from 8.571 kPa (95% CI, 7.685–9.456) at baseline to 6.800 kPa (95% CI, 3.104–10.496) at year 5; however, significance emerged only at year 3 (*p* = 0.035).

When stratified by HCC occurrence, APRI and FIB-4 decreased similarly from baseline to SVR in both subgroups and remained low thereafter (Supplementary Table S2). At most visits, APRI and FIB-4 remained higher in the HCC group than in the non-HCC group.

### 3.4. Risk Factors for Hepatocellular Carcinoma After SVR

To explore predictors of post-SVR HCC after glecaprevir/pibrentasvir therapy, univariate and multivariate Cox models were fitted (Table 4). In univariate analysis, APRI > 1.5 at SVR (*p* = 0.002), FIB-4 > 3.25 at SVR (*p* < 0.001), MELD ≥ 10 (*p* = 0.005), Child–Pugh ≥ 6 (*p* = 0.013), and clinically significant portal hypertension (*p* = 0.018) were associated with HCC. After achieving SVR, neither BMI nor alcohol intake was found to be a significant risk factor for the development of HCC (*p*>0.05). Multivariate analysis identified two independent predictors: FIB-4 > 3.25 (*p* = 0.003) and MELD ≥ 10 (*p* = 0.032). Cumulative incidence of HCC was subsequently stratified by the number of these two risk factors.

**Figure 2:**
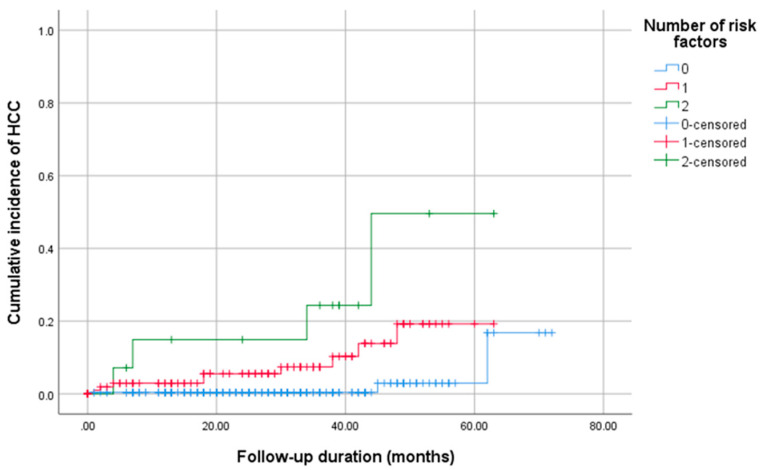
Cumulative incidence of hepatocellular carcinoma according to the number of baseline risk factors identified in the multivariable analysis (FIB-4 > 3.25 at SVR and MELD ≥ 10). Patients were stratified into three groups based on the number of risk factors: 0, 1, or 2.

Based on the multivariable analysis of risk factors for HCC development, namely FIB-4 > 3.25 at SVR and MELD score ≥ 10, patients were stratified into the following three cohorts, as shown in Figure 2: those with no risk factors, one risk factor, or both risk factors. During follow-up, the zero-risk group had the lowest HCC incidence, whereas the single-risk group showed an intermediate incidence. Participants with both risk factors experienced the highest cumulative incidence. This stepwise gradient underscores the additive prognostic value of these factors for post-SVR HCC.

## 4. Discussion

Achieving SVR with direct-acting antivirals has markedly reduced the global burden of HCV-related liver disease. Nevertheless, HCC remains a salient long-term complication, especially among patients with pre-existing fibrosis or functional impairment at the time of viral clearance. In our prospective multicenter cohort, 4.1% of participants developed HCC despite successful glecaprevir/pibrentasvir therapy. Noninvasive fibrosis (FIB-4 > 3.25) and hepatic dysfunction (MELD ≥ 10) measured at SVR independently predicted subsequent HCC. These observations imply that hepatocarcinogenesis can persist after virologic cure, mandating vigilant long-term surveillance of high-risk individuals.

Our findings align with reports showing that HCC risk persists after SVR, especially in advanced fibrosis or cirrhosis [8,13,14]. Prior work indicates that FIB-4 and vibration-controlled transient elastography (VCTE) reliably predict HCC in this population [15]. Because they reflect residual fibrosis or dysfunction, these tools stratify risk and guide post-cure surveillance. Consistent with earlier DAA-era data, advanced age and worsening fibrosis at the time of viral clearance have been associated with a heightened risk of post-SVR HCC, reflecting the compounded impact of biological aging, prolonged hepatic injury, and persistent fibrogenic signaling despite viral eradication [9,16]. Our data extend these observations by showing that combining FIB-4 and MELD adds prognostic precision, confirming that fibrosis regression does not abolish HCC risk.

Unlike earlier cirrhosis-only studies, our cohort spanned the full spectrum of liver disease, broadening applicability. Residual hepatic injury—reflected by elevated FIB-4 and MELD scores—remains a critical determinant of long-term oncologic risk, irrespective of cirrhosis status. Among the predictors, FIB-4 > 3.25 emerged as the most robust. FIB-4, which integrates age, aminotransferase levels, and platelet count, is a validated surrogate for hepatic fibrosis [17]. A value > 3.25 is widely accepted as advanced fibrosis or cirrhosis, conditions that promote hepatocarcinogenesis via chronic inflammation, stellate cell activation, and extracellular matrix remodeling regardless of etiology of chronic liver disease [18].

Likewise, MELD ≥ 10 independently predicted HCC, underscoring the prognostic weight of residual dysfunction despite viral clearance [19,20]. MELD incorporates bilirubin, creatinine, and INR as surrogate measures of synthetic capacity and portal pressure. Persistently elevated MELD after SVR may signal subclinical injury or incomplete recovery, fostering a carcinogenic microenvironment [21]. Metabolic comorbidities—including obesity, diabetes, and alcohol use—continue to drive HCC beyond virologic factors [22]. In our cohort, MELD functioned as a pragmatic surrogate for residual dysfunction, highlighting the influence of non-viral drivers on post-SVR hepatocarcinogenesis.

The persistence of HCC risk after SVR may be partly explained by pathophysiologic processes that remain active beyond viral eradication. Chronic HCV infection induces epigenetic modifications, clonal hepatocyte expansion, and oxidative stress, all of which may sustain oncogenic signaling despite resolution of inflammation [23,24]. Evidence from the activation of hepatic stellate cells and remodeling of the extracellular matrix frequently persists beyond SVR, thereby sustaining a fibrogenic milieu that promotes the development of a pro-tumorigenic microenvironment [25]. Furthermore, unlike interferon-based regimens, DAAs lack intrinsic immunomodulatory and antiproliferative properties, raising the possibility that the rapid decline in viral load achieved by DAAs might alter immune surveillance in a way that permits expansion of pre-existing dysplastic hepatocyte clones [26,27]. However, DAA-treated populations tend to be older, with a higher prevalence of advanced fibrosis, metabolic comorbidities, and prior treatment failure, all of which may contribute to the higher short-term incidence of HCC observed than IFN recipients [28]. After adjusting for these confounders in multivariate analysis, the risks for HCC after DAA and IFN treatments were no different.

This study has several important limitations. First, although patients were followed prospectively, the observational design precludes causal inference between residual hepatic injury and post-SVR HCC. Additionally, unmeasured metabolic, environmental, or genetic factors may have influenced the observed associations. For example, we did not assess the use of antifibrotic co-medications (e.g., statins, antidiabetic agents) or implement lifestyle interventions such as weight-loss counseling or alcohol cessation support, all of which may impact fibrosis regression and HCC risk. Second, although FIB-4 and MELD are noninvasive surrogates of fibrosis and hepatic reserve, they cannot fully capture disease complexity without histology or elastography. Third, the follow-up period captured early but not necessarily late HCC cases, especially in lower-risk groups, underscoring the need for longer surveillance. While the median follow-up was 31 months, HCC occurred at a median of 24 months among affected individuals, and the Kaplan–Meier analysis was extended to 60 months to provide a clearer view of long-term trends. However, since many post-DAA HCC cases may develop after three years, especially in patients with concomitant liver diseases, the current follow-up may not fully capture the long-term risk. Longer follow-up in future studies will be important to confirm these findings and identify delayed HCC cases. Nonetheless, the prospective multicenter design and broad disease spectrum strengthen the study, and the reliance on widely available markers (FIB-4 and MELD) supports their practical value for long-term risk stratification.

## 5. Conclusions

This prospective, multicenter analysis shows that, even after sustained SVR with glecaprevir/pibrentasvir, patients with advanced fibrosis or reduced hepatic reserves remain at risk for HCC. FIB-4 > 3.25 and MELD ≥ 10 at SVR independently predicted tumor development. Because both scores are inexpensive and routinely obtained, and their combined application offers a feasible framework for post-SVR surveillance. Longer follow-up and novel biomarkers are needed to refine risk prediction post-DAA.

## Figures and Tables

**Table 1:** Baseline characteristics at enrollment.

	HCC Occurrence (−), *n* = 379	HCC Occurrence (+), *n* = 16	*p* Value
Male, *n* (%)	168 (44.3)	8 (50.0)	0.655
Age (years)	60.12 ± 12.82	67.80 ± 9.19	0.018
BMI (kg/m^2^)	24.18 ± 3.53	24.00 ± 2.66	0.888
Genotype 1/2/3/6, *n* (%)	133 (35.6)/233 (62.3)/7 (1.9)/1 (0.3)	6 (37.5)/10 (62.5)/0 (0.0)/0 (0.0)	0.949
Significant alcohol intake, *n* (%)	76 (20.2)	4 (25.0)	0.642
Platelet count (10^3^/μL)	187.68 ± 64.95	115.13 ± 53.92	<0.001
AST (IU/mL)	41.22 ± 41.67	49.13 ± 36.15	0.456
ALT (IU/mL)	35.99 ± 50.01	34.44 ± 26.11	0.902
Total bilirubin (mg/dL)	0.74 ± 0.34	1.02 ± 0.52	0.002
Albumin (g/dL)	4.43 ± 2.16	3.99 ± 0.55	0.435
ALBI grade 1/2/3/NA, *n* (%)	303 (86.8)/46 (13.2)/0 (0.0)	12 (80.0)/2 (13.3)/1 (6.7)/1(6.7)	<0.001
Portal hypertension, any sign, *n* (%)	11 (2.9)	3 (18.8)	<0.0001
Cirrhosis, *n* (%)	79 (20.8)	14 (87.5)	—
Child–Pugh score 5/6/7/NA, *n* (%)	354 (95.7)/12 (3.2)/4 (1.1)	9 (60.0)/5 (33.3)/1 (6.7)/1 (6.7)	<0.001
MELD	7.20 ± 2.54	8.92 ± 2.66	0.017
Pre-treatment APRI	1.10 ± 1.42	2.25 ± 2.19	0.027
Pre-treatment FIB-4	3.48 ± 3.66	8.12 ± 6.92	0.017
APRI at SVR12	0.41 ± 0.34	1.09 ± 1.01	0.009
FIB-4 at SVR12	2.38 ± 1.87	5.43 ± 3.56	0.002
LSM (kPa)	8.45 ± 8.46	13.34 ± 7.42	0.075
AFP (ng/mL)	5.27 ± 9.59	49.60 ± 118.26	0.077
Follow-up duration (months)	30.49 ± 17.65	44.87 ± 21.45	<0.001
Pre-treatment HCV RNA (IU/mL)	3,326,704 ± 5,138,275	1,629,454 ± 2,521,693	0.095
HCV recurrence, *n* (%)	1 (0.3)	0 (0.0)	0.837
Mortality, *n* (%)	3 (0.8)	2 (12.5)	<0.001

Data are shown as *n* (%) or mean ± standard deviation. Mann–Whitney U, Chi-square, or Fisher’s exact tests were applied. Abbreviations: HCC, hepatocellular carcinoma; BMI, body mass index; AST, aspartate transaminase; ALT, alanine transaminase; ALBI, albumin–bilirubin; NA, not applicable; MELD, model for end-stage liver disease; APRI, AST-to-platelet ratio index; FIB-4, fibrosis-4; LSM, liver stiffness measurement; AFP, alpha-fetoprotein; HCV, hepatitis C virus.

**Table 2:** Longitudinal changes in liver function scores among patients treated with glecaprevir–pibrentasvir (*n* = 395).

Time Point	Child–Pugh, Mean (95% CI)	*p* Value	MELD, Mean (95% CI)	*p* Value
SVR	5.068 (5.040–5.095)	Ref	7.263 (6.927–7.599)	Ref
1 year	5.055 (5.025–5.085)	0.556	7.414 (6.864–7.713)	0.926
2 years	5.050 (5.015–5.085)	0.443	7.289 (6.864–7.713)	0.926
3 years	5.036 (4.990–5.083)	0.256	7.591 (7.050–8.132)	0.312
4 years	5.015 (4.948–5.082)	0.153	7.252 (6.481–8.023)	0.979
5 years	5.029 (4.936–5.121)	0.427	6.888 (5.807–7.970)	0.517

MELD, model for end-stage liver disease.

**Table 3:** Longitudinal changes in liver fibrosis scores among patients treated with glecaprevir/pibrentasvir (*n* = 395).

	APRI		FIB-4 Index		Liver Stiffness (kPa)	
	Mean (95% CI)	*p* Value	Mean (95% CI)	*p* Value	Mean (95% CI)	*p* Value
Baseline	1.146 (1.068–1.224)	Reference	3.673 (3.424–3.922)	Reference	N/A	N/A
SVR	0.441 (0.363–0.519)	<0.001	2.509 (2.261–2.758)	<0.001	8.571 (7.685–9.456)	Reference
1 year	0.448 (0.364–0.531)	<0.001	2.393 (2.126–2.660)	<0.001	6.828 (5.276–8.381)	0.056
2 years	0.435 (0.339–0.531)	<0.001	2.368 (2.063–2.672)	<0.001	7.371 (4.758–9.985)	0.393
3 years	0.391 (0.272–0.510)	<0.001	2.360 (1.982–2.738)	<0.001	5.352 (2.489–8.215)	0.035
4 years	0.390 (0.231–0.549)	<0.001	2.304 (1.795–2.812)	<0.001	6.129 (3.024–9.235)	0.138
5 years	0.389 (0.168–0.609)	<0.001	2.384 (1.684–3.085)	0.001	6.800 (3.104–10.496)	0.360

APRI, AST-to-platelet ratio index; FIB-4, fibrosis-4 index; SVR, sustained virological response.

**Table 4:** Univariate and multivariate analysis of risk factors for hepatocellular carcinoma after sustained virologic response.

Variable	Univariate Analysis		Multivariate Analysis	
	HR (95% CI)	*p* Value	HR (95% CI)	*p* Value
APRI > 1.5 at SVR	5.534 (1.907–16.056)	0.002	—	—
FIB-4 >3.25 at SVR	9.039 (2.539–32.177)	<0.001	7.102 (1.928–26.159)	0.003
MELD ≥ 10	5.662 (1.709–18.760)	0.005	3.459 (1.112–10.755)	0.032
Child-Pugh score ≥ 6	4.281 (1.353–13.544)	0.013	—	—
Significant portal hypertension	4.836 (1.305–17.923)	0.018	—	—

HCC, hepatocellular carcinoma; SVR, sustained virologic response; HR, hazard ratio; CI, confidence interval.

## Data Availability

All data are included within this article and its Supplementary Materials.

## Supplementary Materials

The following supporting information is available at https://www.mdpi.com/article/10.3390/medicina61091601/s1: Figure S1. Flowchart of the study. Figure S2. Comparison of clinical and biochemical variables between patients with and without hepatocellular carcinoma (HCC) occurrence. Figure S3. (A) Longitudinal changes in liver function and (B) longitudinal changes in liver fibrosis in patients treated with glecaprevir/pibrentasvir, stratified by HCC development. Table S1. Longitudinal changes in liver function scores in patients treated with glecaprevir/pibrentasvir, stratified by HCC. Table S2. Longitudinal liver fibrosis scores in patients treated with glecaprevir/pibrentasvir, stratified by HCC.
