# Supplementary material for: Longitudinal Effects of Glecaprevir/Pibrentasvir on Liver Function, Fibrosis, and Hepatocellular Carcinoma Risk in Chronic Hepatitis C: A Prospective Multicenter Cohort Study
Source: Medicina (Kaunas). 2025 Sep 4;61(9):1601. doi: 10.3390/medicina61091601 (PMC12471995; doi:10.3390/medicina61091601)
Supplement: Supplementary file 1 [file medicina-61-01601-s001.zip › medicina-3744997-supplementary.pdf]

Supplementary Figure S1. Flowchart of the study.

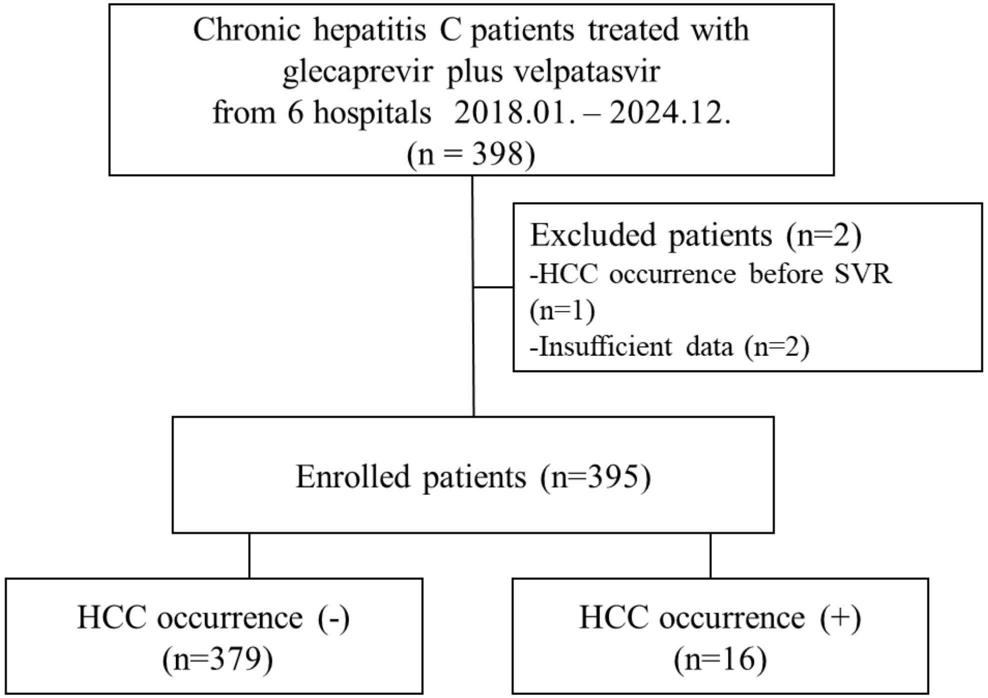

HCC, hepatocellular carcinoma

Supplementary Figure S2. Comparison of clinical and biochemical variables between patients with and without hepatocellular carcinoma (HCC) occurrence.

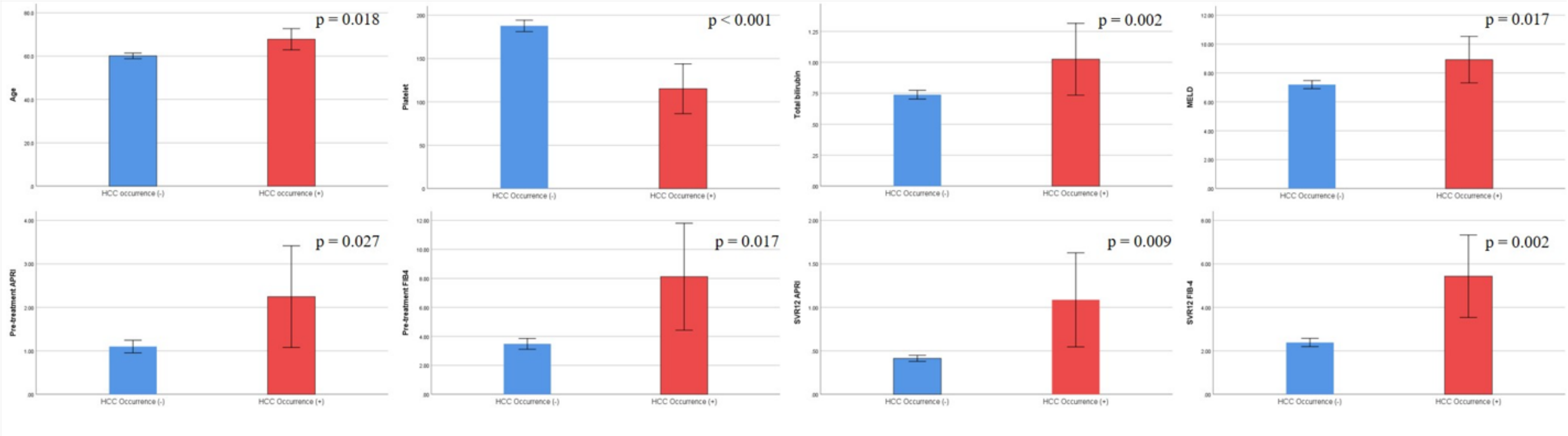

Clustered bar charts illustrate the mean ± standard error of the mean (SEM) for each variable that showed statistical significance ($p < 0.05$) in Table 1. Variables include age, platelet count, total bilirubin, MELD score, pretreatment APRI, pretreatment FIB-4, APRI at SVR12, and FIB-4 at SVR12. Red bars represent patients with HCC occurrence, and blue bars represent those without HCC. Exact p-values from the corresponding statistical tests are overlaid on each panel.

Supplementary Figure S3. **(A)** Longitudinal changes of liver function and **(B)** Longitudinal changes of liver fibrosis in patients who were treated with glecaprevir plus pibrentasvir categorized by development of hepatocellular carcinoma

(A)

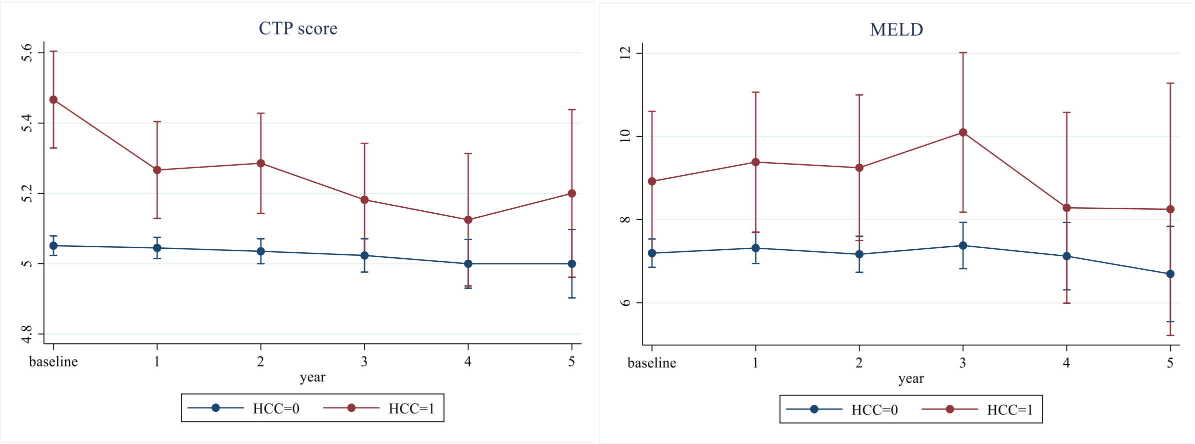

(B)

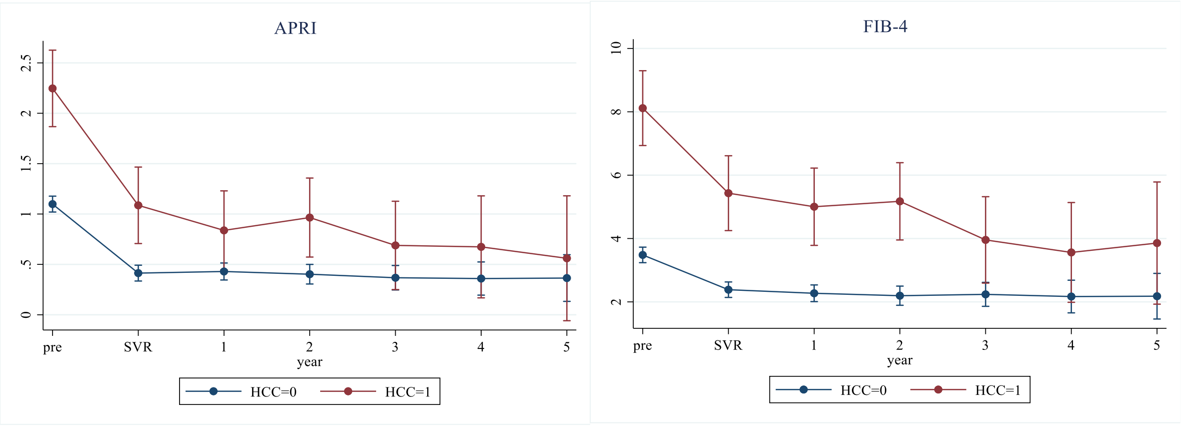

Supplementary Table S1. Longitudinal changes of liver function score in patients who were treated with glecaprevir plus pibrentasvir categorized by HCC occurrence.

	Child-Pugh score			MELD score*		
	HCC occurrence (-) (n=379)	HCC occurrence (+) (n=16)	p-value*	HCC occurrence (-) (n=379)	HCC occurrence (+) (n=16)	p-value*
SVR	5.051 (5.024 – 5.079)	5.467 (5.329 – 5.604)	<0.001	7.195 (6.855 – 7.535)	8.923 (7.240 – 10.606)	0.001
1 year	5.045 (5.015 – 5.075)	5.267 (5.129 – 5.404)	0.001	7.317 (6.942 – 7.691)	9.385 (7.701 – 11.068)	0.002
2 year	5.035 (5.000 – 5.071)	5.286 (5.143 – 5.428)	0.001	7.169 (6.735 – 7.602)	9.250 (7.498 – 11.002)	0.002
3 year	5.024 (4.976 – 5.071)	5.182 (5.021 – 5.342)	0.007	7.379 (6.820 – 7.937)	10.100 (8.181 – 12.019)	0.025
4year	5.000 (4.931 – 5.069)	5.125 (4.937 – 5.313)	0.007	7.123 (6.312 – 7.933)	8.286 (5.992 – 10.580)	0.258
5year	5.000 (4.903 – 5.097)	5.200 (4.962 – 5.438)	0.014	6.694 (5.547 – 7.841)	8.250 (5.216 – 11.284)	0.017
p-value**	0.141			0.951		

Value are presented as mean (95% confidence interval). MELD, model for end-stage liver disease

*, derived from a comparison based on HCC occurrence at each time point, utilizing Wilcoxon rank-sum test

**, derived from linear regression testing temporal changes in each score by HCC occurrence

Supplementary Table S2. Longitudinal changes of liver fibrosis score in patients who were treated with glecaprevir plus pibrentasvir categorized by HCC occurrence.

	APRI			FIB-4 index		
	HCC occurrence (-) (n=379)	HCC occurrence (+) (n=16)	p-value*	HCC occurrence (-) (n=379)	HCC occurrence (+) (n=16)	p-value*
Baseline	1.099 (1.020 – 1.177)	2.247 (1.867 – 2.626)	0.001	3.482 (3.237 – 3.727)	8.116 (6.935 – 9.298)	<0.001
SVR	0.414 (0.335 – 0.492)	1.086 (0.707 – 1.466)	<0.001	2.384 (2.140 – 2.629)	5.432 (4.250 – 6.613)	<0.001
1 year	0.430 (0.345 – 0.514)	0.838 (0.446 – 1.230)	<0.001	2.272 (2.008 – 2.535)	5.004 (3.783 – 6.224)	<0.001
2 year	0.403 (0.306 – 0.500)	0.965 (0.573 – 1.357)	0.001	2.195 (1.892 – 2.498)	5.174 (3.954 – 6.394)	<0.004
3 year	0.368 (0.246 – 0.489)	0.689 (0.250 – 1.127)	0.009	2.238 (1.859 – 2.616)	3.956 (2.592 – 5.320)	0.005
4year	0.360 (0.195 – 0.524)	0.674 (0.168 – 1.180)	0.022	2.169 (1.653 – 2.684)	3.561 (1.986 – 5.136)	0.033
5year	0.365 (0.133 – 0.596)	0.561 (–0.059 – 1.181)	0.088	2.179 (1.458 – 2.900)	3.857 (1.927 – 5.786)	0.019
p-value**	0.049			0.019		

Value are presented as mean (95% confidence interval). APRI, AST to platelet ratio index; FIB-4, Fibrosis-4 index; SVR, sustained virological response

*, derived from a comparison based on HCC occurrence at each time point, utilizing Wilcoxon rank-sum test

**, derived from linear regression testing temporal changes in each score by HCC occurrence
